# Chalcone Synthase in Flavonoid Biosynthesis: Structural Features, Regulatory Mechanisms, and Functions in Plant Defense

**DOI:** 10.3390/cimb48101054

**Published:** 2026-10-13

**Authors:** Rui Liu, Guangzhou Ding, Chunlei Zhao

**Affiliations:** 1 College of Modern Agriculture and Ecological Environment, Heilongjiang University, Harbin 150080, China; 2251948@s.hlju.edu.cn (R.L.); 2001038@hlju.edu.cn (C.Z.); 2 Sugar Beet Engineering Research Center of Heilongjiang Province, Harbin 150080, China

**Keywords:** chalcone synthase, flavonoids, phenylalanine metabolism, type III polyketide synthase, disease resistance defense

## Abstract

Chalcone synthase (CHS, EC 2.3.1.74) is a key entrance enzyme in the biosynthesis of flavonoids from the common phenylpropanoid precursor into a specific flavonoid branch. Beyond its catalytic role, CHS functions as a metabolic hub linking flavonoid production, hormone signaling, and biotic stress defense, and contributes to pigmentation, ultraviolet protection, plant growth, development, and environmental adaptation. Recent structural studies further suggest that chalcone isomerase-like proteins can modulate CHS catalytic efficiency and product specificity, although the physiological significance of this interaction remains incompletely understood across plant species. This review summarizes recent advances in CHS structure and function, with emphasis on metabolic regulation, gene family evolution, disease resistance, and metabolic engineering. Future research should prioritize metabolic flux quantification, functional validation of CHS family members, causal analysis of disease resistance, and precision breeding applications.

## 1. Introduction

Chalcone synthase (CHS, EC 2.3.1.74) is a key enzyme in flavonoid biosynthesis in plants and an important member of the plant type III polyketide synthase superfamily [1]. It produces a variety of flavonoid precursors that play an important role in flowering plants, contributing to the production of anthocyanins, flavonols and proanthocyanidins. The loss of CHS activity will lead to acyanic flowers [2,3]. By enhancing antioxidant capacity and stress defense, CHS helps plants tolerate drought, salinity, low temperature, and pathogen infection while maintaining normal growth and photosynthetic activity [4]. CHS also links flavonoid metabolism to secondary cell wall lignification. In maize, the *Colorless2* (*C2*) gene encodes CHS and contributes to flavonoid biosynthesis in vegetative tissues. Silencing of *C2* reduces tricin incorporation into lignin and alters lignin composition and biomass recalcitrance [5]. CHS is also involved in pollen function, fertilization, seed formation, and fruit quality [6,7]. Altered CHS expression may therefore impair reproductive success and ultimately reduce crop yield. Thus, CHS represents an important regulatory link between plant stress tolerance, reproductive development, and yield formation. 

Flavonoids are among the most important phenolic compounds in plant secondary metabolism and are characterized by a C6–C3–C6 carbon skeleton. They are widely involved in flower coloration, UV protection, antioxidant defense, plant–microorganism interactions, and stress adaptation [8]. Their biosynthesis originates from phenylalanine-derived phenylpropanoid metabolism and is closely connected with malonyl-CoA supply and competing phenylpropanoid branches, including lignin and coumarin biosynthesis. Thus, flavonoid biosynthesis should be viewed not as an isolated pathway, but as part of a broader metabolic network that coordinates carbon allocation, developmental regulation, and environmental responses. 

**Figure 1:**
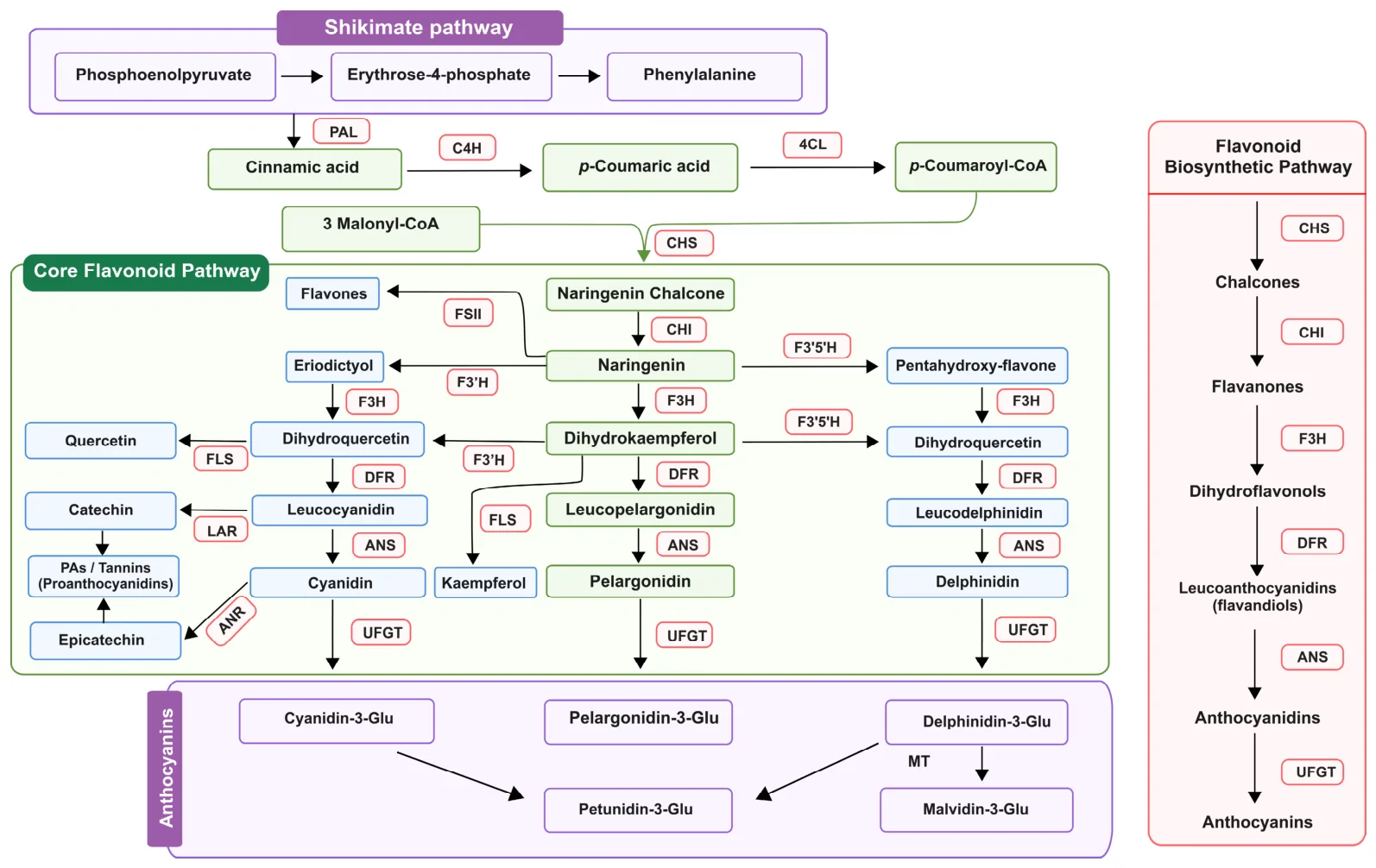
The flavonoid biosynthetic pathway in plants. Abbreviations: PAL, phenylalanine ammonia-lyase; C4H, cinnamate 4-hydroxylase; 4CL, 4-coumarate-CoA ligase; CHS, chalcone synthase; CHI, chalcone isomerase; FSII, Flavone Synthase II; F3H, flavanone 3-hydroxylase; F3′H, flavonoid 3′-hydroxylase; F3′5′H, flavonoid 3′,5′-hydroxylase; FLS, flavonol synthase; DFR, dihydroflavonol 4-reductase; LAR, leucoanthocyanidin reductase; ANS, anthocyanidin synthase; ANR, anthocyanidin reductase; UFGT, UDP-glucose flavonoid 3-O-glucosyltransferase; MT, methyltransferase. Arrows indicate the direction of metabolite conversion in the flavonoid biosynthetic pathway.

CHS is the gateway enzyme that channels common phenylpropanoid precursors into flavonoid biosynthesis. As illustrated in Figure 1, phenylalanine is converted to *p*-coumaroyl-CoA in the canonical pathway through the sequential activities of phenylalanine ammonia-lyase, cinnamate 4-hydroxylase, and 4-coumarate-CoA ligase [9]. CHS then catalyzes the condensation of *p*-coumaroyl-CoA with malonyl-CoA to produce naringenin chalcone, which is subsequently converted by chalcone isomerase into naringenin and further directed into multiple flavonoid branches, including flavonols, anthocyanins, flavanols, and proanthocyanidins [10].

As a member of the type III polyketide synthase family, CHS directly uses CoA ester substrates and functions independently of an acyl carrier protein [11,12,13]. This compact catalytic system enables CHS to integrate upstream substrate availability with downstream flavonoid diversification [14]. Changes in CHS expression, enzymatic activity, subcellular localization, protein interactions, and gene family differentiation can therefore influence total flavonoid accumulation, product composition, and stress-response intensity [15].

The biological significance of CHS extends beyond its catalytic role in flavonoid biosynthesis. Chalcone synthase plays an important role in plant responses to both biotic and abiotic stresses. Pathogen infection induces CHS expression or activity in barley, cucumber, and Norway spruce, accompanied by increased accumulation of flavonoids and other defense-related phenolics [16,17,18]. Suppression of CHS activity nearly abolished induced resistance to powdery mildew in cucumber, whereas co-overexpression of CHS, CHI, and DFR increased flavonoid accumulation and enhanced *Fusarium* resistance in flax [19,20]. CHS genes also respond to abiotic factors such as visible light, UV-B radiation, drought, salinity, and temperature stress, supporting their involvement in stress adaptation through the regulation of flavonoid metabolism [21]. Overall, CHS contributes to plant stress tolerance by regulating the biosynthesis of flavonoids and defense-related secondary metabolites.

Recent advances have shifted CHS research from single-gene expression analysis toward an integrated understanding of enzyme structure, catalytic specificity, metabolic flux, gene family evolution, stress defense, and biotechnological application. This review summarizes current progress in CHS structure and function, with emphasis on metabolic regulation, gene family diversification, disease resistance, and metabolic engineering, and discusses future directions for CHS-based precision breeding and pathway optimization.

## 2. CHS Structure, Catalysis, and Product Specificity

The structure-function relationship of CHS is the basis for understanding its catalytic specificity and metabolic regulation [22]. As a typical type III polyketide synthase, CHS accommodates aromatic acyl-CoA starter substrates and malonyl-CoA extender units within a conserved catalytic framework. Its active-site architecture controls chain extension, intermediate stabilization, and final cyclization, thereby determining substrate preference, product type, and enzymatic efficiency [23,24]. This means that changes in a few key amino acids of CHS may affect substrate selection, product type, and enzymatic efficiency.

### 2.1. Protein Structure Characteristics of CHS

The CHS monomer is usually assembled by α-helix and β-sheet, and forms a relatively conservative active center [25]. The conserved Cys-His-Asn catalytic triad is the core of the acyl transfer and condensation reaction of CHS, in which the cysteine residue is responsible for receiving the initial acyl group and forming the acyl enzyme intermediate, and the histidine and asparagine are involved in stabilizing the intermediate state of the reaction [26].

In addition to the catalytic triad, the volume, polarity and entrance channel around the active cavity also affect the substrate adaptation [27]. The CHS protein sequences of different species are generally conserved, but there are differences in the N-terminal, substrate channel and surface interaction regions [28]. These differences may correspond to tissue-specific expression, stress induction ability or the ability to form complexes with other flavonoid enzymes.

**Figure 2:**
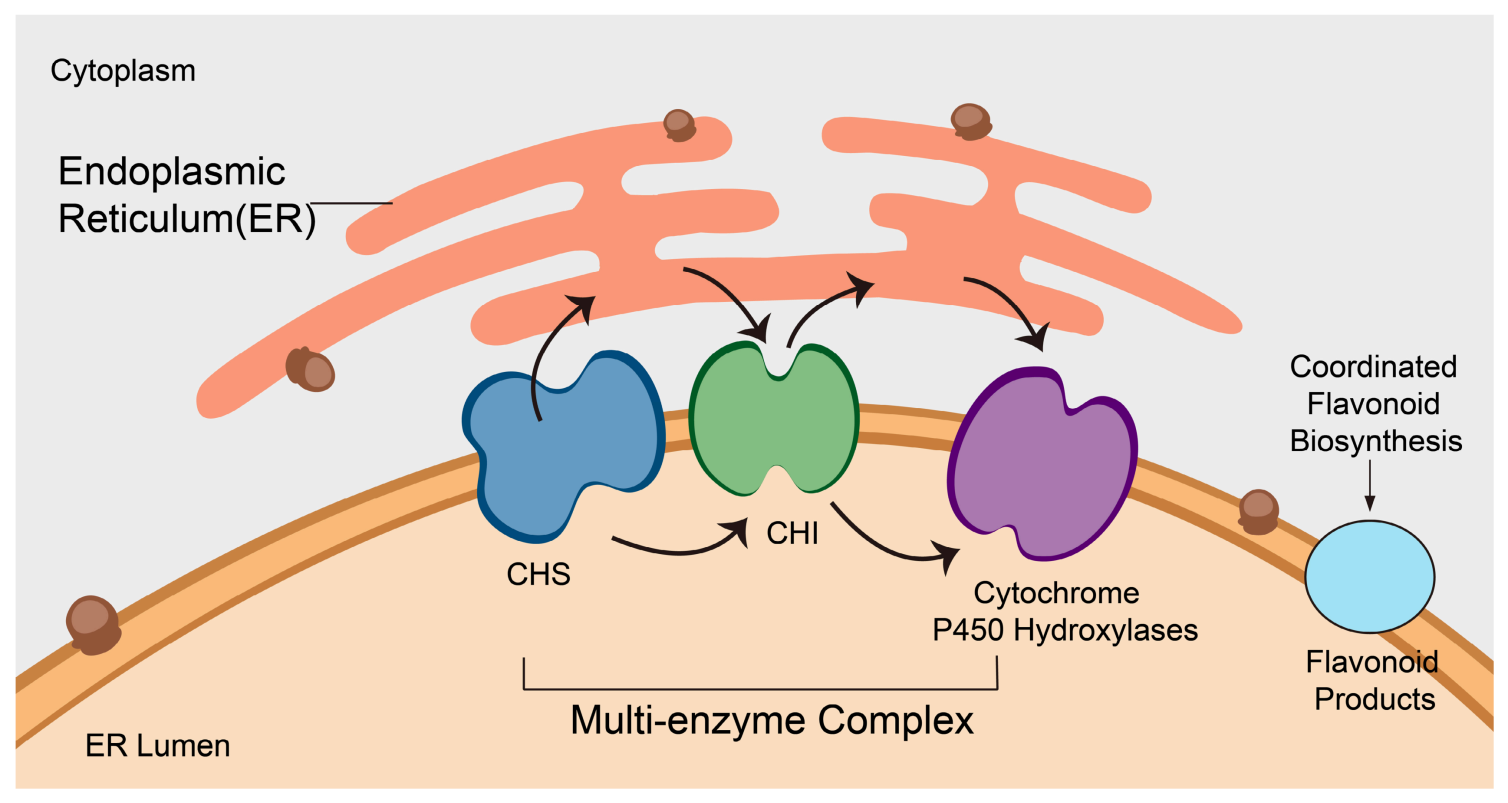
Subcellular organization and substrate channeling in CHS-mediated flavonoid biosynthesis. CHS associates with CHI and ER-associated cytochrome P450 hydroxylases to form a local multi-enzyme complex that promotes chalcone formation, downstream modification, and efficient substrate channeling during flavonoid biosynthesis.

CHS is usually localized in the cytoplasm, but flavonoid biosynthesis may not occur in a fully homogeneous cellular environment. Increasing evidence suggests that flavonoid enzymes may function within local microdomains or multi-enzyme complexes associated with the cytoplasm or endoplasmic reticulum surface. The spatial organization among CHS, CHI, and cytochrome P450 hydroxylases may improve substrate transfer efficiency and contribute to coordinated flavonoid accumulation (Figure 2) [29,30].

### 2.2. Catalytic Mechanism and Substrate Specificity

The CHS catalytic reaction generally includes initial substrate loading, three rounds of malonyl-CoA decarboxylative condensation, tetraketone intermediate formation, and Claisen-type cyclization [31]. The final product naringenin chalcone is a direct substrate of downstream CHI. If CHI activity is insufficient, chalcone may also undergo non-enzymatic isomerization or be utilized by other modifying enzymes [32,33].

The catalytic performance of CHS depends not only on transcript abundance but also on enzyme activity, substrate availability, cellular microenvironment, and coordination with downstream enzymes [34]. Hence, CHS function should be evaluated by integrating enzyme kinetics, metabolomics, protein interaction analysis, and isotope-tracing approaches rather than relying solely on gene expression data [35].

Although *p*-coumaroyl-CoA is the classical starter substrate of CHS, CHS or CHS-like enzymes from different plant species may also show varying degrees of adaptability to cinnamoyl-CoA, caffeoyl-CoA, feruloyl-CoA, and related aromatic acyl-CoA substrates [36]. This substrate flexibility contributes to the diversity of chalcone and flavonoid derivatives and provides potential targets for enzyme engineering. However, broadened substrate acceptance may also increase product complexity, especially when downstream enzymes cannot efficiently process the corresponding intermediates [37].

### 2.3. CHS–CHIL Complex and Functional Regulation

The catalytic specificity of CHS is modulated by interactions with associated proteins, particularly chalcone isomerase-like proteins (CHILs). Unlike canonical chalcone isomerases (CHIs), CHILs generally lack enzymatic isomerase activity but promote flavonoid biosynthesis by regulating CHS function [38]. This CHS–CHIL interaction indicates that CHS product specificity is governed not only by its intrinsic active-site architecture, but also by protein-mediated regulation of substrate binding, CoA release, and stabilization of catalytic residues.

The CHS–CHIL complex provides a mechanistic basis for coordinated control of chalcone formation and downstream flavonoid biosynthesis [34]. By optimizing the catalytic microenvironment of CHS and enhancing substrate turnover efficiency, CHIL reduces non-specific intermediate accumulation and directs metabolic flux toward specific flavonoid branches [39]. These findings extend the functional view of CHS from a standalone catalytic enzyme to a protein-interaction-centered metabolic module.

### 2.4. Relationship with Other Type III Polyketide Synthases

CHS is closely related to other type III polyketide synthases, such as stilbene synthase (STS), benzalacetone synthase (BAS), aloesone synthase (AUS), and other CoA-dependent polyketide-forming enzymes. These enzymes share a conserved catalytic framework, including similar catalytic residues and CoA-binding channels, but differ in active-site cavity size, residue composition, and cyclization patterns [40]. Such structural variations determine their substrate preferences and product profiles, leading to the formation of chalcones, stilbenes, benzalacetones, aloesones, and other polyketide derivatives.

The comparison between CHS and related type III polyketide synthases provides an important basis for understanding the evolutionary diversification of plant secondary metabolic enzymes. Although these enzymes may originate from similar ancestral catalytic scaffolds, subtle amino acid substitutions around the active-site cavity can redirect reaction routes and generate distinct metabolites [41]. Therefore, the structural similarity and functional divergence between CHS and other type III polyketide synthases are important for explaining CHS family evolution, catalytic specificity, and potential enzyme engineering applications.

## 3. CHS and Associated Metabolic Pathways

The metabolic significance of CHS lies in its role as a key metabolic node. Its catalytic activity not only depends on the upstream supply of starter substrates but also determines the initial metabolic flux entering downstream flavonoid branches. Accordingly, CHS should not be regarded merely as a structural gene in the flavonoid biosynthetic pathway. Its function is closely associated with phenylpropanoid metabolism, malonyl-CoA supply, CoA activation, intracellular transport, and metabolic compartmentalization.

### 3.1. Association Between CHS and Upstream Phenylpropanoid Metabolism

The upstream phenylalanine metabolism provides the aromatic acyl starting substrate for CHS [42]. The activity of PAL, C4H and 4CL determines the ability of *p*-coumaroyl-CoA formation, and *p*-coumaroyl-CoA is also an important precursor of lignin, coumarin and other phenylpropanoid products [43]. Thus, the up-regulation of CHS is often accompanied by the induction of upstream genes such as PAL and 4CL, which reflects the overall trend of plants to enhance phenylpropanoid metabolism under stress conditions [44].

However, an increase in the phenylalanine pathway does not necessarily mean an increase in flavonoids. If the carbon flow preferentially enters the lignin or cell wall phenolic acid cross-linking, the substrate available for CHS may be limited by competition [45]; on the contrary, if the regulatory network promotes the co-expression of CHS, CHI and flavonoid branching enzymes, more *p*-coumaroyl-CoA enters flavonoid synthesis.

In the experimental design, to determine the degree of coupling between CHS and the upstream pathway, the expression of key genes in PAL, C4H, 4CL, CHS, CHI and lignin branches can be simultaneously detected [46], and the contents of metabolites such as *p*-coumaric acid, *p*-coumaroyl-CoA, naringenin, quercetin or anthocyanin can be combined [47]. If only the total flavonoids were detected, it was easy to cover up the changes in the direction of different branches; if only CHS expression is detected, it is impossible to determine whether the substrate actually enters the flavonoid pathway.

### 3.2. Coordination Between CHS and Malonyl-CoA Supply

Malonyl-CoA is an essential three-carbon extension unit in each catalysis of CHS, and it is also an important substrate for fatty acid elongation, wax and other polyketide metabolism [48]. Hence, the catalytic efficiency of CHS is influenced by the size of intracellular malonyl-CoA pool, subcellular distribution and competition of different metabolic processes. 

In metabolic engineering, increasing CHS expression is often not enough to significantly increase the yield of target flavonoids. It is necessary to simultaneously enhance malonyl-CoA supply, optimize initial substrate formation, reduce bypass consumption, and coordinate downstream conversion steps [49]. Studies on microbial synthesis of flavonoids have also shown that substrate pool engineering and enzyme cascade balance are often more critical than simple overexpression of CHS [37,50].

In plant cells, the local availability of malonyl-CoA may be more important than the total amount. Fatty acid synthesis occurs mainly in plastids, and many flavonoid synthases are located in the cytoplasm or related to the endoplasmic reticulum. The compartment transport, local production and competitive consumption of malonyl-CoA may affect the CHS reaction [51]. Therefore, to accurately increase flavonoid production in the future, attention must be paid to the constraints imposed by acetyl-CoA carboxylase, malonyl-CoA synthesis and transport, and cell compartmentalization on the supply of CHS substrates.

### 3.3. Relationship Between CHS and Downstream Flavonoid Branch

The CHS product naringenin chalcone forms naringenin through CHI and enters multiple downstream branches [52]. F3H makes it enter the dihydroflavonol branch, FLS guides flavonols, DFR and ANS guide anthocyanins, and products such as isoflavones, flavonoids and proanthocyanidins can also be formed in specific species [53]. Thus, CHS governs metabolic input flux, while downstream enzymes and regulatory networks dictate final product type.

Recent studies on CHI-like proteins have further changed the understanding of downstream relationships. CHIL protein itself may lack typical isomerase activity, but it can improve the efficiency of flavonoid synthesis by interacting with CHS or CHI, suggesting that plants may reduce the loss of intermediate diffusion through multi-enzyme complexes or metabolic channels [54].The study of structural interaction also supports that the spatial organization of CHS and CHI/CHIL will affect the efficiency of flavonoid biosynthesis [55].

### 3.4. Competition and Coordination Between CHS and Other Phenylpropanoid Branches

The flavonoid synthesis pathway in which the CHS is located is not isolated, but shares the upstream carbon flow and some CoA activation precursors with lignin, stilbenes, coumarins, lignans, and other phenylpropane branches. The change in CHS activity not only affects the synthesis of flavonoids, but also may change the carbon flow distribution between different branches of phenylpropanoid metabolism [56]. Under pathogen infection conditions, plants often need to accumulate small molecules such as flavonoids, coumarins, and lignans with antioxidant, antibacterial, or signal regulation effects, and also need to form physical barriers through lignin deposition, phenolic acid cross-linking, and cell wall thickening [57]. Accordingly, how *p*-coumaroyl-CoA and its related upstream precursors are distributed between flavonoid branches and cell wall-related branches will directly affect the type and intensity of plant disease resistance strategies.

This branch competition relationship demonstrates that simply increasing CHS expression does not necessarily only bring positive effects. On the one hand, up-regulation of CHS may promote the accumulation of flavonoids, improve the ability of plants to scavenge reactive oxygen species, regulate signal transduction and limit pathogen expansion. On the other hand, if the flavonoid branch consumes a large amount of limited carbon source or CoA activation precursor, it may also affect the synthesis of lignin, phenolic acid or other structural defense substances, resulting in a certain metabolic cost [58]. In some tissues or developmental stages, there may be a trade-off between increased flavonoid accumulation and growth, quality or cell wall formation. In other cases, CHS upregulation may coincide with lignin deposition and cell wall reinforcement, jointly strengthening plant pathogen restriction capacity.

Consequently, the evaluation of the physiological function of CHS cannot be judged only by its expression level. A more reasonable idea is to combine CHS expression, flavonoid metabolite accumulation, lignin or phenolic acid changes, cell wall structure changes, and disease resistance phenotypes [59]. Especially in the study of disease resistance, the role of CHS may change due to different pathogen types, infection stages, tissue parts and plant development status [60]. If CHS-mediated flavonoid synthesis is mainly responsible for antioxidant and signal regulation functions, it may complement the lignin branch; if the two compete for the same finite precursor, there may be a trade-off in resource allocation between different defense strategies [61]. This also explains why CHS upregulation varies across pathogens and tissues: it may indicate enhanced disease resistance, or merely activation of phenylpropanoid metabolism unrelated to improved resistance.

## 4. Characteristics of CHS Gene Family in Different Species

With the development of plant genome sequencing and comparative genomics, CHS research has expanded from a single model plant to a large number of economic crops and non-model species [62]. Genome-wide analyses reveal marked interspecific divergence in CHS gene copy number, chromosomal localization, gene architecture and expression profiles, offering key clues to flavonoid metabolic diversity [63].

### 4.1. Expansion and Evolution of CHS Gene Family

Cross-species studies have shown that CHS and CHS-like genes continue to expand during the evolution of plants from aquatic to terrestrial [64]. Related studies have identified a large number of CHS and CHS-like genes in 178 species, suggesting that the expansion of the CHS family is related to plant adaptation to terrestrial ultraviolet light, pathogenic stress, and complex ecological interactions [62].

The differences in the number of CHS members in different species may be due to genome-wide replication, tandem duplication, fragment replication and transposition-related events. Studies on buckwheat [65], Chinese cabbage [66], maize [10], cherry [67], and bamboo [68] demonstrate that replication events can produce multiple CHS members, and then the members are differentiated in promoter elements, expression patterns and protein structures (Table 1).

From the perspective of selection pressure, the core catalytic sites of CHS are often subjected to purification selection, and the new members produced by replication are more likely to accumulate and mutate in promoters, non-conservative regions and expression regulation. This model is conducive to maintaining the basic catalytic ability while generating new expression scenarios, such as one member maintaining pigment synthesis in floral organs, and another member responding to pathogen infection in roots or leaves [69].

**Table 1:** The identification and functional study of CHS gene family in different plant species.

Species	CHS Gene Number	Protein Length (aa)	Function	References
*Fagopyrum tataricum* (L.)	14 FtCHS	344–395	Flower-enriched expression; likely regulates floral development/coloration.	[65]
*Phyllostachys edulis*	17 PeCHS	309–405	PeCHS14 is stress-inducible and a potential stress-resistance candidate gene.	[68]
*Chrysanthemum nankingense*	16 CnCHS	197–401	CnCHS10 likely functions in floral flavonoid synthesis and pigmentation.	[70]
*Glycine max* (L.) Merr.	14 GmCHS	341–392	Functions in isoflavone, flavonoid, and phytoalexin biosynthesis; tissue-specific expression.	[71]
*Brassica rapa* L. ssp. *pekinensis*	10 BrCHS	388–396	BrCHS2/8 respond to MeJA, salt, and drought; BrCHS4 regulates leaf flavonoids and anthocyanins.	[66]
*Zea mays* L.	68 ZmCHS	300–398	ZmCHS25 supports salt tolerance, flavonoid biosynthesis, and osmotic adjustment.	[10]
*Cerasus pseudocerasus* L.	3 CpCHS	PavCHS: 261–391; CpCHS1: NA	CpCHS1 enhances drought resistance and oxidative stress tolerance.	[67]
*Saussurea involucrata*	10 SiCHS	286–401	SiCHS genes respond to low-pressure stress, with reduced flavonoid accumulation and gene expression.	[72]
*Solanum melongena* L.	7 SmCHS	327–396	SmCHS4/5/6/7 respond to heat stress and regulate anthocyanin accumulation and peel color.	[73]
*Dendrobium catenatum*	14 DcCHS	349–504	DcCHS genes show tissue-specific expression and respond to drought, heat, cold, and salt stress.	[74]
*Oryza sativa* L.	27 OsCHS	393–431	OsCHS genes are broadly expressed in rice tissues and involved in salt/drought stress responses.	[75]

NA: The length range of CHS protein (amino acid) in this species was not clearly reported in the original literature.

### 4.2. CHS Gene Structure and Conserved Motifs

Most CHS genes have relatively conserved exon-intron structures and protein conserved motifs, especially the catalytic triad and its surrounding regions are highly conserved [76]. This conservation indicates that the basic catalytic function of CHS is constrained by strong selection, and the core structure is not prone to destructive mutations.

At the same time, there were significant differences in non-core regions, promoter cis-acting elements and intron lengths among different CHS members. Studies on cucumber [77] and bread wheat [78] reveal that light response, hormone response, MYB binding, stress response and pathogen-related elements were common in CHS promoter, which provided a molecular basis for its differential expression in different tissues and stress conditions (Table 2).

Gene structure analysis can also be used to determine whether CHS members may undergo pseudogenization or new functionalization. If a member lacks a key catalytic site, has an abnormal premature termination codon, or has a very low expression, it needs to be carefully determined that it still has CHS function [72]. On the contrary, if a member retains the complete catalytic motif and is strongly expressed in specific tissues or under stress, it is more worthy of priority for functional verification.

### 4.3. Expression Patterns and Functional Differentiation of CHS Genes

The biological roles of CHS genes are closely associated with their spatial and temporal expression patterns as well as the functional differentiation of individual family members. Because CHS is an enzyme rather than a specialized metabolite, its “distribution” cannot be described simply according to its abundance in different organs. Instead, CHS distribution should be considered at multiple biological levels, including the occurrence and copy number variation in CHS homologs among plant lineages, the expression of individual family members in different organs and tissues, and changes in CHS transcript abundance, protein abundance, and enzyme activity across developmental stages, genotypes, and environmental conditions.

#### 4.3.1. Expression of CHS Genes in Different Tissues and Organs

Although CHS genes are phylogenetically widespread, individual family members frequently exhibit pronounced tissue and organ specificity. In cereals, CHS genes show member-specific expression in both vegetative and reproductive tissues. Rice OsCHS transcripts have been detected across multiple organs and developmental stages, whereas maize ZmCHS genes generally exhibit low expression in seeds, leaves, endosperm, and embryos, with ZmCHS01 and ZmCHS02 showing comparatively broad expression during seed and leaf development [75,81].

In legumes, root-biased CHS expression is particularly prominent and is associated with the production of flavonoids and isoflavonoids involved in rhizobial signaling, nodulation, and defense. In pea, CHS1 and CHS3 are expressed in both petals and roots, whereas CHS2 expression is largely restricted to roots [82]. Soybean CHS family members are expressed in roots, stems, leaves, floral organs, pod walls, seed coats, and developing embryos, but individual genes display distinct tissue preferences. CHS7 and CHS8, for example, show relatively high expression in roots, while specific CHS loci are differentially regulated in developing seed coats [71] (Table 3).

Tissue-specific CHS expression is also evident in pigmentation-related organs. In the colored leaves of cannabis (*Cannabis sativa*), CHS and other flavonoid-pathway genes are closely associated with the accumulation of anthocyanins and related phenolic compounds [83]. In fruit crops, CHS expression is frequently enriched in epidermal tissues or increases during fruit coloration and ripening. CHS protein accumulates mainly in grape berry skin, leaves, shoot tips, and stem phloem. In tomato, fruit ripening is accompanied by increased CHS activity and flavonoid accumulation in epidermal and cuticular tissues [6]. Ripening-related CHS expression has also been demonstrated in strawberry fruit, where CHS silencing strongly decreases anthocyanin accumulation. In highbush blueberry, several CHS genes, particularly members of one phylogenetic subfamily, are preferentially expressed during the red, purple, and blue stages of fruit ripening [22].

However, fruit-associated expression is not a universal feature of all CHS family members. In mango, many MiCHS genes show relatively high expression in roots and seeds, whereas only a subset is strongly expressed in fruit peel [84]. In citrus, three functionally expressed CHS genes show distinct patterns among roots, stems, cotyledons, and leaves, and their expression can be modified by methyl jasmonate treatment [85]. These findings indicate that the spatial expression patterns of CHS family members reflect both organ specialization and responses to developmental or environmental cues.

**Table 3:** Distribution and expression of CHS genes in representative plant tissues.

Species and CHS Genes	Tissue	References
*Pisum sativum*: CHS1, CHS2, CHS3 *Glycine max*: GmCHS7, GmCHS8 *Mangifera indica*: MiCHS2, MiCHS4, MiCHS5, MiCHS7, MiCHS9, MiCHS11, MiCHS16, MiCHS17, MiCHS20 *Citrus* spp.: CitCHS1–CitCHS3	Root.	[71,82,84,85]
*Zea mays*: ZmCHS01, ZmCHS02 *Glycine max*: GmCHS8 *Manihot esculenta*: multiple MeCHS genes *Vitis vinifera*: CHS protein	Leaf	[71,81,86,87]
*Citrus* spp.: CitCHS genes *Glycine max*: GmCHS7, GmCHS8 *Vitis vinifera*: CHS protein *Manihot esculenta*: multiple MeCHS genes	Stem and phloem	[71,85,86,87]
*Pisum sativum*: CHS1, CHS3 *Glycine max*: GmCHS7, GmCHS8 *Mangifera indica*: multiple MiCHS genes	Flower and petal	[71,82,84]
*Zea mays*: ZmCHS01, ZmCHS02 *Glycine max*: GmCHS7, GmCHS8 *Mangifera indica*: MiCHS2, MiCHS4, MiCHS5, MiCHS11, MiCHS16, MiCHS17	Seed and embryo	[71,81,84]
*Mangifera indica*: MiCHS2, MiCHS4, MiCHS5, MiCHS16, MiCHS19 *Vitis vinifera*: CHS protein *Solanum lycopersicum*: SlCHS	Fruit peel, skin and epidermis	[6,84,87]
*Vaccinium corymbosum*: VcCHS8, VcCHS13, VcCHS21 *Fragaria × ananassa*: FaCHS; *Solanum lycopersicum*: SlCHS	Ripening fruit	[6,22,88]

#### 4.3.2. Developmental Stage, Genotype, and Environment Related Expression

CHS expression changes substantially during organ development. In sweet cherry, three CHS genes are progressively downregulated during flower-bud differentiation, whereas their expression during fruit ripening differs between cultivars [67]. In highbush blueberry, VcCHS8, VcCHS13, and VcCHS21 are expressed more strongly during colored fruit stages than during the green-fruit stage [22]. Similar developmental regulation has been observed in tomato, strawberry, grapevine, and other fruit crops, where increased CHS expression generally coincides with epidermal flavonoid and anthocyanin accumulation.

Environmental conditions can further modify CHS expression. In *Ginkgo biloba*, UV-B treatment increases leaf flavonoid accumulation and is accompanied by activation of the flavonoid pathway [89]. In plum (*Prunus salicina* ‘Friar’), fruit flesh turns red during cold storage, accompanied by altered expression of anthocyanin biosynthetic genes, including CHS [90]. These observations indicate that CHS expression is not only developmentally regulated but also responsive to environmental stimuli.

Genotype-dependent variation is especially evident in tissues in which visible pigmentation is closely associated with CHS activity [91,92]. Differences among soybean seed-coat genotypes, sweet cherry cultivars, and fruit-color genotypes of horticultural crops indicate that variation in CHS expression may arise from structural gene differences, promoter activity, transcriptional regulation, or gene-silencing mechanisms. Therefore, CHS expression patterns should be interpreted together with developmental stage, genotype, and environmental conditions.

#### 4.3.3. Functional Differentiation and Regulatory Diversification of CHS Family Members

Expression patterns provide important clues to CHS function, but expression evidence alone cannot establish functional differentiation. Functional divergence among CHS family members has been reported in several plant species, particularly in species containing duplicated or homologous CHS genes. In gerbera (*Gerbera hybrida*), duplicated CHS genes exhibit functional differentiation in anthocyanin biosynthesis, indicating that different CHS members may contribute differently to flavonoid production in floral organs and pigment-accumulating tissues [93]. Such divergence suggests that expansion of the CHS gene family does not necessarily result in complete functional redundancy but may instead be accompanied by specialization among individual members.

CHS function is also influenced by regulatory mechanisms beyond transcription. In petals of *Paeonia* ‘He Xie’, CHS can undergo ubiquitination-mediated degradation through interaction with a RING-H2 protein, indicating that CHS activity is affected by protein stability and post-translational regulation [94]. Studies on flower-color fading in *Syringa oblata* likewise suggest that changes in anthocyanin accumulation should be interpreted in combination with developmental regulation, transcriptional control, and functional validation rather than gene expression alone [95].

Many genome-wide studies remain largely based on bioinformatic prediction and qRT-PCR validation, with limited support from mutants, overexpression lines, complementation assays, or metabolic flux analyses [96,97]. Accordingly, future studies should move beyond the identification and expression profiling of CHS family members toward direct functional characterization of individual genes. Particular attention should be given to distinguishing CHS genes that play major roles in flavonoid biosynthesis, those involved in stress responses, and CHS-like members that may have undergone functional diversification.

## 5. CHS in Plant Development and Stress Defense

The physiological function of CHS is mainly reflected by its product flavonoids, but the effects of different flavonoid branches are not the same. Anthocyanins and flavonols are often associated with pigment, antioxidant and UV protection. Isoflavones and some chalcones play a prominent role in legume defense and symbiosis. Proanthocyanidins and phenolic acid derivatives may be involved in herbivore and cell wall defense [98,99].

### 5.1. Roles in Pigmentation, UV Protection, and Antioxidant Metabolism

CHS is an important upstream enzyme for pigment formation in flowers, fruits and seed coats [100,101]. Anthocyanin accumulation usually requires the synergistic expression of structural genes such as CHS, CHI, F3H, DFR, ANS and UFGT, and is regulated by transcription factors such as MYB-bHLH-WD40 complex [102]. When CHS expression is reduced or its function is impaired, the entry flux into the flavonoid pathway decreases, thereby limiting anthocyanin biosynthesis [103].

In horticultural plants, CHS is often used to explain flower color variation and fruit coloring differences. A study in apple (*Malus domestica*) demonstrated that the expression patterns of CHS family members across different tissues and developmental stages were correlated with flavonoid accumulation [104]. However, the color phenotype is not determined by CHS alone, and downstream hydroxylation, glycosylation, acylation and vacuolar transport also affect the final pigment stability.

Pigment formation studies also suggest that CHS regulation has significant spatiotemporality [105]. The activation of CHS in the early stage of petal development can provide a substrate for subsequent anthocyanin accumulation, while the synergistic expression of CHS with glycosyltransferase and transporter in the fruit ripening stage affects the stable deposition of pigment [106]. Therefore, if CHS is used as a target for flower or fruit color improvement in breeding, it is necessary to consider the expression initiation time, tissue specificity and downstream modification ability, rather than only pursuing high expression of the whole plant.

Flavonoids have the ability to absorb UV-B, scavenge reactive oxygen species and stabilize the redox state of cells. Hence, CHS plays a fundamental role in plant response to strong light, ultraviolet and oxidative stress [107,108]. The light signal can promote the expression of CHS and other flavonoid structural genes through regulatory factors such as HY5 and MYB, thereby increasing the accumulation of flavonols and anthocyanins [109].

Under drought, salt, heavy metal and pollutant stresses, CHS-induced expression is often associated with flavonoid accumulation and enhanced antioxidant capacity [110]. These flavonoids may act as direct antioxidant molecules or indirectly enhance stress resistance by regulating ROS signaling, protective membrane systems, or chelating harmful ions [111]. However, the role of CHS under abiotic stress is mainly a background function in this review, whereas the following sections focus more on its significance in disease resistance and defense responses.

The antioxidant effect of flavonoids also intersects with disease resistance. Pathogen infection is often accompanied by ROS outbreak; moderate ROS is conducive to defense signal transmission, and excessive ROS causes cell damage [112]. CHS-promoted flavonoid accumulation may help plants reduce oxidative damage while maintaining ROS signals, making defense responses more stable. However, if flavonoids excessively scavenge ROS, it may also weaken the defense process partially dependent on ROS, which requires further study in combination with pathogen type and time dynamics.

### 5.2. CHS-Mediated Defense Against Biotic Stress

Phenylpropanoid metabolism and flavonoid metabolism are often rapidly activated after plants are infected by fungi, bacteria or viruses. The induced expression of CHS means that plants may direct more carbon flow to chalcones, flavonols, isoflavones or other defensive phenols to limit pathogen expansion or regulate defense signals [113]. The antibacterial mechanism of flavonoids may include destroying the membrane structure of pathogens, affecting nucleic acid or protein synthesis, inhibiting virulence factors, and changing the redox environment around pathogens [114].

**Figure 3:**
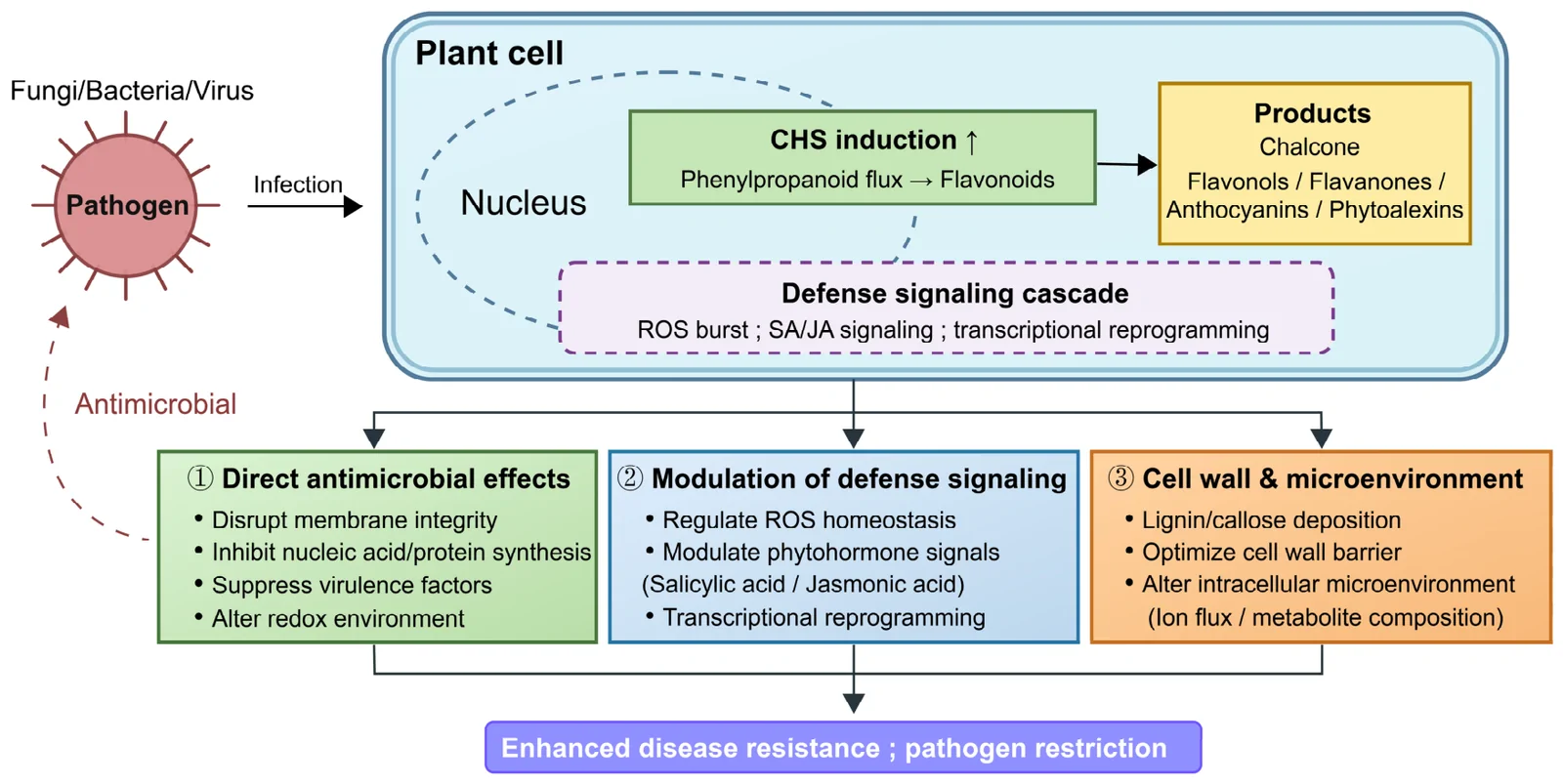
The pathways of CHS-mediated flavonoid route in plant defense. Solid arrows indicate regulatory relationships or sequential biological processes, whereas the dashed arrow indicates the antimicrobial effect of flavonoids on pathogen restriction.

From the perspective of defense level, CHS-mediated flavonoid pathway may affect disease resistance through at least three pathways [115] (Figure 3). The secondary metabolites synthesized by pathway can directly exert antibacterial activity and inhibit the proliferation and infection of pathogenic bacteria. At the same time, it can regulate the plant reactive oxygen species homeostasis, endogenous hormone signal and transcriptional regulation system, and coordinate the activation of the overall defense response; it can also reshape the flow of phenylpropanoid metabolites, optimize the cell wall structure barrier, and change the intracellular microenvironment that is not conducive to pathogen colonization and infection. These three effects commonly coexist during plant–pathogen interactions, so layered experimental verification is required and all defensive responses cannot be simply ascribed to flavonoid antibacterial activity [7].

#### 5.2.1. Pathogen Infection Induced CHS Expression

Pathogen infection-induced CHS expression is a common phenomenon in plant defense responses [116]. Studies on cucumber and other CHS families have shown that some members have obvious responses to biotic stress, suggesting that there may be a defense-specific division of labor among CHS family members [77].

It should be noted that pathogen-induced CHS expression can only indicate its involvement in the defense process but cannot directly demonstrate a causal role in disease resistance. To establish a causal relationship between CHS function and disease resistance, gene silencing or knockout and overexpression approaches should be combined with measurements of lesion area, pathogen biomass, and flavonoid content, together with complementation assays where appropriate.

#### 5.2.2. CHS-Mediated Accumulation of Flavonoids Defense Substances

CHS promotes the accumulation of defensive metabolites by increasing the flux of flavonoid entrance [117]. Flavonols, chalcones, isoflavones and some phenolic acid derivatives can directly inhibit the growth of pathogens, and can also act as signal molecules to affect SA, JA, ET and other defense pathways [118]. In specific species, CHS upregulation may promote the accumulation of phytoalexin-like substances or antitoxins, forming a chemical defense line [119].

This defense has species and pathogen specificity [120]. Isoflavonoids are more prominent in leguminous plants, while cruciferous and rosaceous plants may show synergistic accumulation of flavonols, anthocyanins or other phenylpropanoid products [1]. On this basis, the up-regulation of ‘CHS’ cannot be equated with the enhanced resistance of all flavonoids, and the target species, pathogen types, and downstream product profiles must be specifically analyzed [121].

#### 5.2.3. CHS and Cell Wall Enhancement and Metabolic Redistribution

The disease resistance process depends not only on soluble antimicrobial substances, but also on structural defenses such as cell wall strengthening, lignification, and callose deposition [122]. Since CHS and branches such as lignin share phenylpropanoid precursors, there may be competition between flavonoid accumulation and cell wall enhancement after pathogen infection, or they may occur synergistically due to the overall enhancement of the upstream pathway [21].

If CHS promotes flavonoid accumulation and reduces oxidative damage, cell wall defense may obtain a more stable cell environment. If the carbon flow excessively turns to flavonoids and weakens lignin synthesis, it may affect the formation of structural barriers [123]. Accordingly, analyzing the disease resistance function of CHS requires incorporating soluble flavonoids, lignin, cell wall phenolic acid crosslinking, and ROS dynamics into the same framework [46].

### 5.3. CHS and the Formation of Symbiotic Signaling Molecules

Flavonoids are also an important signal for the interaction between plants and symbiotic microorganisms, especially in the symbiosis of legume rhizobia [124]. Specific flavonoids secreted by roots can induce the expression of nod gene in rhizobia, thereby initiating the nodulation signal process [125]. Since CHS controls the entrance of flavonoid synthesis, its expression status affects the supply of rhizosphere signal molecules [126].

There is a certain intersection between symbiotic signals and disease resistance defense [127]. Plants need to maintain a balance between promoting beneficial microbial interactions and inhibiting pathogen infection. Flavonoids may either recruit symbiotic bacteria or act as antibacterial substances or signals to regulate defense responses [128]. This makes CHS an important entry point for understanding the selectivity of plant-microorganism interactions [129].

From an ecological point of view, the flavonoid signal involved in CHS reflects the selective recognition of microorganisms by plants [130]. The same flavonoid molecule in the rhizosphere may induce some beneficial microorganisms and inhibit some pathogenic or competitive molecular mechanism underlying regulation disease resistance, but also change rhizosphere microbial community composition and plant long-term adaptability [131]. In the future, the combination of CHS function research and rhizosphere microbiome may reveal the broader role of flavonoid metabolism in plant ecological defense.

## 6. Regulatory Mechanism and Application Prospect of CHS

The expression of CHS is controlled by multiple factors such as developmental stage, tissue type, light, hormone, pathogen signal and epigenetic status [132]. Because CHS is located at the entrance of flavonoid synthesis, regulating its expression is often used by plants to rapidly change flavonoid flux, but the final metabolic outcome depends on whether the entire pathway is synergistic [133].

### 6.1. Transcriptional Regulation of CHS

The CHS promoter often contains MYB, bHLH, light response and stress response related cis-elements, indicating that its transcriptional regulation is highly plastic [134]. The MYB-bHLH-WD40 complex is a classic module that regulates anthocyanin and some flavonoid branches. Many MYB factors can directly or indirectly activate structural genes such as CHS, CHI, F3H, and DFR [135].

Recent studies illustrate that specific MYB factors can directly activate CHS members. For example, AcMYB176 can activate AcCHS5 and regulate flavonoid biosynthesis [136], and FtMYB45 is also closely related to flavonoid accumulation [137]. These results suggest that CHS family members are often incorporated into species-specific transcriptional regulatory networks, and different members may respond differently to the same transcription factor [138].

Epigenetic regulation may also be involved in the expression of CHS and downstream anthocyanin genes. Histone acetylation, deacetylation and chromatin state can change the binding efficiency of transcription factors, thus affecting the expression of flavonoid structural genes [139]. In the future, the combination of transcriptome, ATAC-seq, ChIP-seq and metabolomics will help to analyze the true level of CHS expression regulation.

### 6.2. Regulation of CHS by Hormones and Pathogen Signals

**Figure 4:**
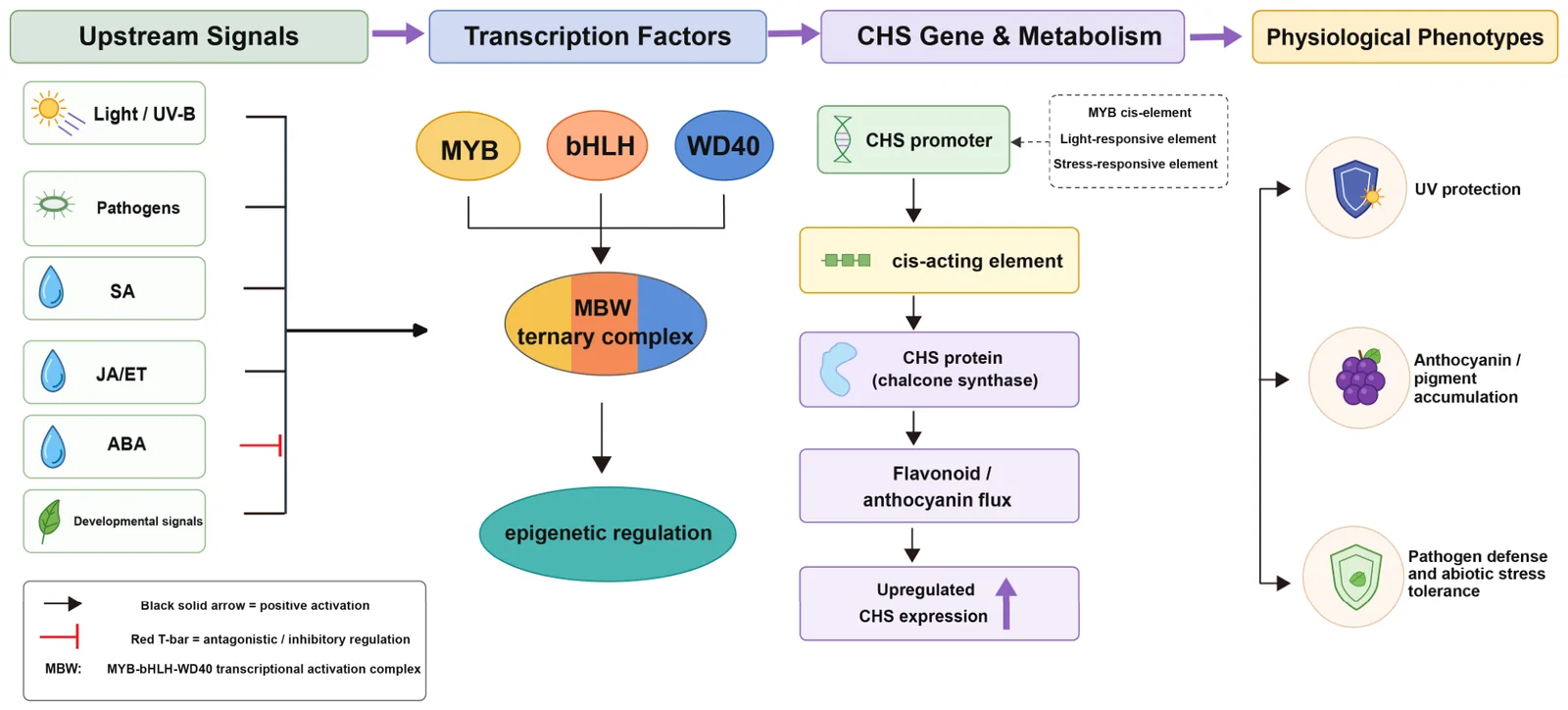
Regulatory network of CHS transcription in flavonoid biosynthesis modulated by develop mental, environmental, and epigenetic signals.

Hormone signals such as SA, JA, ET, and ABA are closely related to CHS expression [140] (Figure 4). In pathogen infection, SA is often associated with anti-biotrophic pathogens, JA/ET is more often involved in anti-necrotrophic pathogens and injury responses, and flavonoid metabolism can interact with these signals [141]. The regulation of JA and ET signaling on flavonoid synthesis suggests that CHS may be at a junction between defense hormones and secondary metabolism [142].

ABA and stress signals can also affect flavonoid metabolism [143]. Some studies indicate that ABA is involved in the regulation of flavonoid or anthocyanin accumulation and is associated with drought, salt and maturation processes [144]. Under pathogen stress, ABA exerts antagonistic or synergistic interactions with SA and JA signals, necessitating hormone network analysis to clarify CHS induction patterns [145].

Furthermore, light signals and developmental peptide signals can also regulate flavonoid synthesis [146]. Light-induced CHS expression is a common mechanism for plants to improve UV protection and anthocyanin accumulation. Studies on the involvement of developmental signals such as CLEL6 in anthocyanin regulation indicate that CHS-related pathways are also related to growth and development regulation [147].

The regulation of CHS by pathogenic signals may also be affected by pathogenic life forms [148]. Biotrophic pathogens usually induce SA-related defense. Necrotrophic pathogens and insect feeding are more likely to activate JA/ET-related pathways, and different hormone combinations may have different effects on CHS and downstream branches [149]. The disease resistance function of CHS depends on specific pathogen systems. It is better to compare the differential expression and metabolic results of CHS family members induced by different pathogens in the same plant.

### 6.3. Application of CHS in Disease Resistance Breeding and Metabolic Engineering

In the application of disease resistance breeding and metabolic engineering, CHS can be used as candidate genes, molecular markers and flavonoid metabolic regulation nodes [150]. However, its application value should not be judged only based on pathogen-induced expression, but should be comprehensively evaluated in combination with gene overexpression, knockout or promoter editing, flavonoid metabolite accumulation, pathogen biomass changes, and growth, yield and quality traits [151].

As a key entrance enzyme in the flavonoid synthesis pathway, the function of CHS depends on the synergistic effect of upstream precursor supply, downstream structural enzymes and transcriptional regulatory network [152]. Thus, in crop disease resistance improvement, CHS, PAL/4CL, CHI, FLS, DFR, MYB/bHLH transcription factors and cell wall defense-related genes should be included in the regulatory network for analysis. In metabolic engineering, it is necessary to simultaneously optimize the supply of *p*-coumaroyl-CoA and malonyl-CoA, product conversion efficiency and metabolic toxicity mitigation mechanism [153]. Gene editing techniques such as CRISPR-Cas provide an effective tool for analyzing the functions of CHS family members and accurately regulating flavonoid metabolism. Nevertheless, due to the functional redundancy and metabolic compensation of CHS family, single-gene manipulation may be difficult to obtain stable phenotypes [154]. In general, CHS is more suitable as a modular regulatory node in the flavonoid metabolism and disease resistance defense network, rather than an isolated improvement target; the system design based on multi-gene synergistic regulation and overall optimization of metabolic flux is more helpful to improve disease resistance and flavonoid yield while reducing growth cost and metabolic bottleneck.

## 7. Conclusions

This review summarizes current knowledge of the structural characteristics, evolutionary diversification, expression patterns, and biological functions of CHS genes in plants. As the key entry enzyme of flavonoid biosynthesis, CHS connects phenylpropanoid substrate supply with downstream flavonoid accumulation and defense-related metabolism. Its conserved type III PKS catalytic structure, functional differentiation among gene family members, and coordination with CHI/CHIL collectively influence chalcone formation, metabolic flux, and flavonoid product specificity. Through this complex regulatory network, CHS contributes to pigmentation, UV tolerance, antioxidant protection, symbiotic signaling, and resistance to biotic and abiotic stresses, suggesting its potential as a candidate gene for crop improvement and flavonoid metabolic engineering.

However, the available evidence remains insufficient to fully explain how individual CHS members coordinate enzyme activity, tissue-specific expression, stress signaling, and metabolic partitioning. Because CHS is positioned at an upstream branch point of the phenylpropanoid pathway, its manipulation may alter the balance among lignin, anthocyanins, flavonols, and other phenylpropanoid-derived metabolites. Future studies should therefore integrate genetic analysis, enzyme characterization, subcellular localization, protein interactions, metabolic flux analysis, and stress phenotyping. Several questions remain to be clarified: How do different CHS members contribute to specific flavonoid products and stress responses? Which regulatory proteins and metabolic enzymes form functional complexes with CHS? How does CHS manipulation reshape carbon allocation among competing phenylpropanoid branches? Can target traits be improved without compromising plant growth and ecological adaptability? Addressing these questions will deepen our understanding of CHS-mediated metabolic network remodeling and facilitate its effective application in coordinating plant growth, quality formation, and defense.

## Figures and Tables

**Table 2:** Promoter cis-acting elements of CHS gene family in different plant species.

Species	Number of Exons	Characteristics of Promoter Cis-Acting Elements	Intron/Gene Structure Characteristics	References
*Arachis hypogaea*	2–3 (53% for 2, 47% for 3)	Promoters are enriched in HRE, STRE, and LRE; tetraploids show higher total element numbers but similar mean elements per promoter.	CHS genes mostly have 2 exons and 1 intron, or 3 exons and 2 introns. STS gene structure is highly conserved.	[79]
*Cucumis sativus* L.	2	Hormone/stress/growth/light-responsive cis-elements in 1.5 kb promoters.	It belongs to the smaller CHS family, and the members are distributed on different chromosomes.	[77]
*Nicotiana tabacum*	2	Conserved cis-elements for drought, light, hormone, and pollen-specific responses.	All five genes possess 2 exons and 1 intron. The splicing sites of these introns comply with the GT-AG rule, and intron lengths differ between distinct gene members.	[80]
*Triticum aestivum* L.	2	Promoter divergence with MYB/MYC, CHS-Unit 1, DRE, and HSE elements drives stress-related functional differentiation.	The 1185 bp coding region is highly conserved. Genes locate on chromosome 2 short arm of Chs-1 and long arm of Chs-4 homologous groups.	[78]

## Data Availability

No new data were created or analyzed in this study. Data sharing is not applicable to this article.
